# Sex assigned at birth may modify health‐related quality of life in children treated with peanut oral immunotherapy

**DOI:** 10.1111/pai.70177

**Published:** 2025-08-22

**Authors:** Sophie A. Rosser, Melanie Lloyd, Paxton Loke, Sarah Ashley, Michael D. O'Sullivan, Patrick Quinn, Michael Gold, Mimi L. K. Tang

**Affiliations:** ^1^ Allergy Immunology Murdoch Children's Research Institute Parkville Victoria Australia; ^2^ School of Population and Global Health University of Melbourne Parkville Victoria Australia; ^3^ Department of Paediatrics University of Melbourne Parkville Victoria Australia; ^4^ National Allergy Centre of Excellence (NACE) Parkville Victoria Australia; ^5^ Centre for Medicine Use and Safety Monash University Parkville Victoria Australia; ^6^ Department of Allergy and Immunology The Royal Children's Hospital Parkville Victoria Australia; ^7^ Monash Children's Hospital Monash Health Clayton Victoria Australia; ^8^ Immunology Department Perth Children's Hospital, Child and Adolescent Health Service Nedlands Western Australia Australia; ^9^ Discipline of Paediatrics, Medical School The University of Western Australia Perth Western Australia Australia; ^10^ Telethon Kids Institute The University of Western Australia Nedlands Western Australia Australia; ^11^ Department of Paediatrics, Adelaide Medical School University of Adelaide Adelaide South Australia Australia; ^12^ Women's and Children's Hospital Adelaide North Adelaide South Australia Australia

**Keywords:** effect modification, food allergy, oral immunotherapy, quality of life

## Abstract

**Background:**

The high burden of peanut allergy underscores the need for treatment options that improve patient health‐related quality of life (HRQL). However, the modifying effect of sex assigned at birth on treatment‐related outcomes remains poorly understood. We sought to investigate whether sex modifies treatment effect on the change in overall and subdomain HRQL during the PPOIT‐003 trial.

**Methods:**

PPOIT‐003 was a multicenter, randomized controlled trial in 201 children with peanut allergy (aged 1–10) allocated to combined probiotic and peanut oral immunotherapy (PPOIT), peanut oral immunotherapy alone (OIT), or placebo. Participant HRQL was measured with the Food Allergy Quality of Life–Parent Form (FAQLQ‐PF) at baseline, end‐of‐treatment, and 12 months post‐treatment. Multivariable linear regression with an interaction term was used to investigate the relationship between treatment and HRQL in males (*N* = 128, 63.68%) and females (*N* = 73, 36.32%).

**Results:**

Sex‐modification of total FAQLQ‐PF scores was present between baseline to end of 12 month follow‐up (*p* = .008). In this time, improvement in FAQLQ‐PF scores was significantly better in active compared to placebo groups for males (PPOIT vs. Placebo: −1.003 [95% CI: −1.571, −0.436]; OIT vs. Placebo: −1.250 [95% CI: −1.805, −0.695]), but not for females where improvement also occurred in the placebo group (PPOIT vs. Placebo: −0.148 [95% CI: −0.914, 0.617]; OIT vs. Placebo: 0.252 [95% CI: −0.547, 1.052]). Separate analysis of study phases revealed sex effect modification was greater during treatment than during post‐treatment follow‐up in domains related to food anxiety (*p* = .037) and emotional impact of allergy (*p* = .063).

**Conclusion:**

Sex modifies HRQL outcomes during peanut OIT. Greater understanding of the biological and psychosocial drivers of post‐treatment HRQL will facilitate personalized management approaches.

**Abbreviations**
CONSORT: Consolidated Standards of Reporting Trials
DBPCFC: Double‐blind placebo‐controlled food challenge
EI: Emotional impact
FA: Food‐related anxiety
FAQLQ‐PF: Food Allergy Quality of Life Questionnaire–Parent Form
HRQL: Health‐related quality of life
IgE: Immunoglobulin E
LRT: Likelihood ratio test
OIT: Oral immunotherapy
PPOIT: Probiotic and peanut oral immunotherapy
PPOIT‐003: Probiotic peanut oral immunotherapy versus oral immunotherapy and placebo in children with peanut allergy in Australia, randomized phase 2b trial
SDL: Social and dietary limitations
SES: Socioeconomic status
T0: Baseline
T1: End of immunotherapy treatment, 18 months post‐baseline
T3: 12‐month post‐trial follow‐up, 30 months post‐baseline

**Key message**

Personalized treatment options are needed to address HRQL impairment caused by IgE‐mediated peanut allergy, but the role of patient characteristics in shaping treatment outcomes is poorly understood. We identify sex as a potentially important treatment effect modifier of HRQL. Sex differences were greater during active treatment than during post‐treatment follow‐up, driven by food anxiety and emotional impact HRQL subdomain scores. Our findings indicate more research into personalized approaches to patient care that explore sex differences in treatment response is needed to provide benefit to patients.

## 1 INTRODUCTION

The greatest psychosocial impact on an individual from food allergies is a reduced health‐related quality of life (HRQL), particularly amongst children and their caregivers.^1^, ^2^, ^3^, ^4^ We previously conducted a systematic review that observed HRQL differences between males and females over the course of immunotherapy treatment.^5^ However, limited reporting in included studies prevented any conclusions regarding which sex experienced more HRQL benefit during treatment. Greater understanding of whether and how sex modifies treatment effects on HRQL outcomes will inform personalized approaches to food allergy treatment.

Poorer HRQL and psychological wellbeing have been observed in individuals with food allergy compared to non‐allergic populations and those with other chronic health conditions, including diabetes and rheumatic diseases.^6^, ^7^, ^8^, ^9^ For allergies with a high lifetime risk of allergic reaction, such as persistent peanut allergy, these reductions in HRQL have been attributed to the stress and lifestyle restrictions associated with avoidance management practices.^10^, ^11^, ^12^ Importantly, there are differences in the burden of food allergy between males and females, with strong evidence that females with food allergy experience poorer HRQL than males in a non‐interventional setting.^5^ Currently, food allergy treatment and management options are applied broadly to all patients without personalization that accounts for sex differences. These practices do not support, and may instead undermine, personalized allergy management.

Efforts have been directed to finding active treatment options that reduce reactions to accidental ingestion and improve HRQL in a personalized manner, with quality of life having recently been identified as a critical core outcome in food allergy studies.^13^ One such treatment option—oral immunotherapy (OIT)—has become routinely available for desensitization of peanut allergy in the United States, Canada, and parts of Europe.^14^, ^15^, ^16^ Its potential as a remission treatment continues to be investigated in clinical trials, including the Australian PPOIT‐003 multicenter, randomized control trial.^1^ Emerging therapies that induce remission are reported to improve HRQL by removing the need for indefinite maintenance dosing and decreasing the need for rigorous avoidance diets.^2^, ^17^ Previous analysis of PPOIT‐003 data found that both the combined probiotic and peanut OIT (PPOIT) and peanut OIT alone (OIT) were effective at inducing remission and improving HRQL compared to placebo, with the addition of probiotic adjuvant providing a safety benefit that was most significant in preschool‐aged children.^1^ Sex assigned at birth, age, and history of anaphylaxis have since been identified as potentially important modifiers of treatment effect on HRQL in the PPOIT‐003 cohort.^18^ In relation to sex modification of treatment‐related HRQL improvement, major knowledge gaps remain regarding whether sex impacts HRQL during or after treatment and the psychosocial constructs that are driving differential treatment effect on HRQL. This study aims to investigate when (during active therapy or in the short‐term post‐treatment) and how sex modification of the effect of OIT on HRQL occurs.

## 2 METHODS

### 2.1 Study design, participants, and intervention allocation

A post hoc analysis was conducted using data collected as part of the PPOIT‐003 trial in accordance with CONSORT guidelines. The PPOIT‐003 trial is a three‐armed, multicenter, blinded, superiority trial; ethics approval was provided by the Royal Children's Hospital Human Research Ethics Committee (HREC 35246) and the Child and Adolescent Health Service Perth (RGS 2543). The protocol and primary outcomes have been previously published (Australian New Zealand Clinical Trials Registry 12,616,000,322,437).^1^

Briefly, 201 children with peanut allergy were recruited from three tertiary Australian hospitals (in Adelaide, Melbourne and Perth), stratified (by study site, age, peanut skin prick test) and randomized 2:2:1 to receive an 18‐month course of PPOIT, OIT alone, or placebo, respectively. Eligibility criteria were: 1–10 years of age, body weight of at least 7 kg, IgE‐mediated peanut allergy confirmed by failed double‐blind placebo‐controlled food challenge (DBPCFC) and positive peanut skin prick test or peanut‐specific serum‐IgE at screening. Written informed consent was obtained from parents or guardians of the participant. Treatment efficacy (achievement of remission, desensitization or persisting allergy) was assessed via two exit DBPCFCs, one conducted at the end of the 18‐month course of treatment (T1) and the other after an avoidance period of 8 weeks.

### 2.2 Quality of life outcomes

**FIGURE 1:**
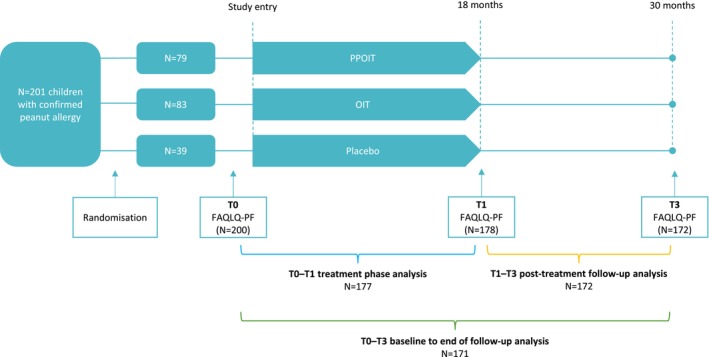
Study flow diagram. Showing key time points of FAQLQ‐PF measurement and number of participants included in the three key analyses of treatment, post‐treatment follow‐up, and baseline to end of follow‐up periods. Participants were included in analyses if FAQLQ‐PF scores were available for both time points included in corresponding change score calculations. FAQLQ‐PF, Food Allergy Quality of Life Questionnaire–Parent Form; OIT, oral immunotherapy; PPOIT, probiotic and peanut oral immunotherapy; T0, study baseline; T1, end‐of‐treatment; T3, 12‐month follow‐up.

Caregiver proxy‐report of child HRQL was measured at three timepoints—at baseline (T0), end‐of‐treatment (T1), and 12‐months post‐treatment (T3) using the Food Allergy Quality of Life Questionnaire–Parent Form (FAQLQ‐PF) (Figure 1). Participants remained blinded to their treatment allocation throughout; however, they became aware of their allergy status during the exit DBPCFCs. Total HRQL score and subscores quantifying the emotional impact of food allergy (EI), food anxiety in relation to allergen avoidance and exposure risk (FA), and food allergy‐related social and dietary limitations (SDL) were examined.^19^

FAQLQ‐PF scores were calculated in accordance with the FAQLQ‐PF scoring manual. Participants with missing data for 20% or more of the relevant FAQLQ‐PF questions were excluded from score calculations and subsequent analyses. FAQLQ‐PF change scores were calculated as mean differences in Total scores and subscores (EI, FA, and SDL) for three periods of interest for use in later regression models.

A minimum clinically important decrease of 0.45–0.50 points, previously established for the FAQLQ‐PF, was used to interpret HRQL differences between time points, sexes, and treatment groups.^19^, ^20^

### 2.3 Data analysis

In this analysis, we defined participant sex according to sex assigned at birth. Results were stratified and reported according to the Sex and Gender Equity in Research (SAGER) guidelines.^21^

Analysis was conducted using Stata version 18 (StataCorp LLC, TX) according to a complete‐case approach. Multivariate linear regression models were constructed to assess interaction effects between treatment allocation and sex, and their associations with change in FAQLQ‐PF total and subscores. To isolate treatment phase impacts from post‐treatment changes in HRQL, the sex‐modified relationship between treatment and FAQLQ‐PF change between T0 to T1 (the treatment period) was compared with that between T1 to T3 (the post‐treatment period). Regression models included adjustment for baseline HRQL to mitigate the effects of any potential baseline imbalance.

Likelihood ratio tests (LRTs) assessing the goodness of fit of two models, one with and one without inclusion of sex‐treatment interaction, were performed to provide further evidence of effect modification presence or absence.

Sex differences in individual FAQLQ‐PF items were also descriptively explored using mean changes in item scores between T0 and T3 for each treatment arm to determine whether particular items were more influential in driving aggregate score results.

### 2.4 Investigation of bias

Sensitivity analysis comparing models with and without adjustment for baseline HRQL were conducted to assess the effects of baseline imbalance in HRQL on the change in total score and subscores between T0 and T3 (Table S1). Presence of selection bias was descriptively explored, including comparison of the proportions of participants that completed the FAQLQ‐PF at each time point by treatment allocation (Table S2).

## 3 RESULTS

Participant characteristics were similar between treatment arms and sexes (Table 1). The proportion of males and females allocated to each treatment was similar, although there were more male (*N* = 128) than female (*N* = 73) participants overall. Most participants had concomitant eczema (64.29%–78.57%), and a history of anaphylaxis to peanut was reported in 22.58%–39.29% across treatment groups. Little diversity was seen in the socioeconomic status of participants. Most participants allocated PPOIT or OIT achieved remission (sustained unresponsiveness) outcomes at the end of treatment (T1), whether male (PPOIT: 39.58%, OIT: 49.09%) or female (PPOIT 54.84%, OIT 53.57%). This was not seen in the placebo arm, where only one male and one female participant passed both the end‐of‐treatment DBPCFC and the second DBPCFC after 8 weeks of avoidance.

**TABLE 1:** Characteristics of male and female children (*N* = 201) randomized to PPOIT, OIT, and placebo.

Characteristic	PPOIT arm (*N* = 79)		OIT arm (*N* = 83)		Placebo arm (*N* = 39)	
	Males (*N* = 48)	Females (*N* = 31)	Males (*N* = 55)	Females (*N* = 28)	Males (*N* = 25)	Females (*N* = 14)
Age (years) – mean (sd)	5.93 (3.00)	6.20 (2.96)	5.82 (2.61)	5.76 (2.85)	6.09 (2.62)	5.75 (2.88)
History of anaphylaxis – *N* (%)						
To peanut	15 (31.25)	7 (22.58)	18 (32.73)	11 (39.29)	7 (28.00)	5 (35.71)
To other food	8 (16.67)	9 (29.03)	11 (20.00)	3 (10.71)	4 (16.00)	4 (28.57)
Other current food allergies^a^ – *N* (%)						
Yes	26 (54.17)	16 (51.61)	21 (38.28)	15 (53.57)	17 (68.00)	10 (71.43)
No	22 (45.83)	15 (48.39)	34 (61.82)	13 (46.43)	8 (32.00)	4 (28.57)
Concomitant allergic conditions – *N* (%)						
Asthma	10 (20.83)	8 (25.81)	21 (38.18)	10 (35.71)	10 (40.00)	4 (28.57)
Eczema	37 (77.08)	23 (74.19)	39 (70.91)	22 (78.57)	18 (72.00)	9 (64.29)
Socioeconomic status^b^ – *N* (%)						
1	3 (6.25)	1 (3.23)	3 (5.45)	1 (3.57)	3 (12.00)	0 (0.00)
2	6 (12.50)	4 (12.90)	6 (10.91)	1 (3.57)	2 (8.00)	2 (14.29)
3	5 (10.42)	2 (6.45)	5 (9.09)	6 (21.43)	3 (12.00)	2 (14.29)
4	12 (25.00)	6 (19.35)	18 (32.73)	6 (21.43)	2 (8.00)	2 (14.29)
5	17 (35.42)	15 (48.39)	21 (38.18)	11 (39.29)	15 (60.00)	7 (50.00)
Baseline FAQLQ‐PF score^c^ – median (IQR)	2.17 (1.12–3.31)	1.58 (0.77–3.42)	1.71 (0.87–3.42)	1.43 (0.70–2.18)	2.36 (1.14–3.69)	2.88 (1.36–4.30)
Baseline eliciting dose – *N* (%)						
80 mg	7 (14.58)	5 (16.13)	10 (18.18)	1 (3.57)	5 (20.00)	2 (14.29)
160 mg	10 (20.83)	6 (19.35)	10 (18.18)	2 (7.14)	6 (24.00)	5 (35.71)
320 mg	7 (14.58)	6 (19.35)	15 (27.27)	9 (32.14)	7 (28.00)	1 (7.14)
640 mg	14 (29.17)	7 (22.58)	7 (12.73)	3 (10.71)	2 (8.00)	1 (7.14)
1250 mg	5 (10.42)	4 (12.90)	5 (9.09)	6 (21.43)	4 (16.00)	3 (21.43)
2500 mg	5 (10.42)	3 (9.68)	8 (14.55)	7 (25.00)	1 (4.00)	2 (14.29)
Post‐treatment clinical allergy outcome^d^ – *N* (%)						
Remission (SU)	19 (39.58)	17 (54.84)	27 (49.09)	15 (53.57)	1 (4.00)	1 (7.14)
Desensitization	14 (29.17)	11 (35.48)	16 (29.09)	3 (10.71)	0 (0.00)	0 (0.00)
Allergic	15 (31.25)	3 (9.67)	12 (21.82)	10 (35.71)	24 (96.00)	13 (92.86)

Abbreviations: FAQLQ‐PF, Food Allergy Quality of Life Questionnaire–Parent Form; IQR, interquartile range; OIT, oral immunotherapy; PPOIT, probiotic and peanut oral immunotherapy; SU, sustained unresponsiveness.

^a^ Participants may have had multiple other current food allergies; percentages do not add to 100%.

^b^ Represented as Socio‐Economic Indexes for Areas (SEIFA) quintiles of disadvantage.^47^ A score of 1 represents most disadvantaged, 5 represents least disadvantaged. Data only available for 187 participants (121 males and 66 females); proportion of those with data available per treatment arm presented, percentages do not add to 100%.

^c^ The FAQLQ–PF is inversely scaled: higher scores reflect poorer HRQL.

^d^ Determined via two exit DBPCFCs (5 g cumulative peanut protein). First challenge conducted at the end of the 18‐month course of treatment; the second challenge at 8 weeks post‐treatment with peanut elimination in the intervening period. Failure of the first DBPCFC = Allergic; Pass first challenge, fail second challenge = Desensitization (no remission); Pass both challenges = Remission.

**FIGURE 2:**
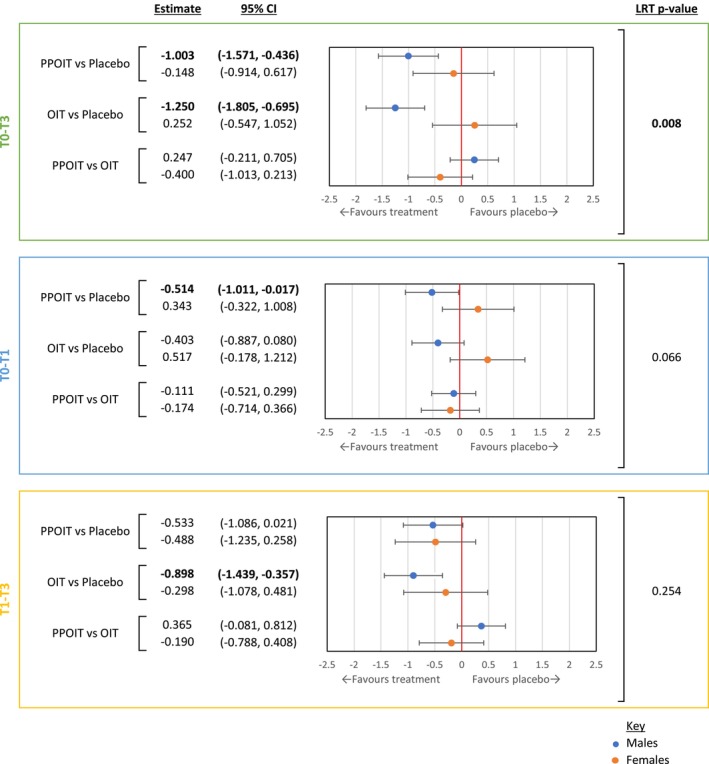
Change in FAQLQ‐PF total scores in baseline to end of follow‐up (T0 to T3), treatment (T0 to T1) and post‐treatment follow‐up (T1 to T3) analyses between treatment groups for males and females. Clinically and statistically significant mean FAQLQ‐PF change score estimates and significant LRT *p*‐values are bolded. The FAQLQ‐PF is inversely scaled: Higher scores reflect poorer HRQL. The T0 to T3 sex‐HRQL interaction for total FAQLQ‐PF scores has been previously published.^18^ FAQLQ‐PF, Food Allergy Quality of Life Questionnaire–Parent Form; HRQL, health‐related quality of life; LRT, likelihood ratio test; OIT, oral immunotherapy; PPOIT, probiotic and peanut oral immunotherapy.

Evidence for effect modification by sex was present in FAQLQ‐PF total scores across the full analysis period from T0 to T3 (*p* = .008), and was stronger during the treatment period (T0 to T1) (*p* = .066) than the post‐treatment follow‐up period (T1 to T3) (*p* = .254) (Figure 2). In the full analysis period (T0 to T3), significant improvements in FAQLQ‐PF total scores were observed in males receiving PPOIT or OIT compared to males receiving placebo (PPOIT vs. Placebo: −1.003 [95% CI: −1.571, −0.436]; OIT vs. Placebo: −1.250 [95% CI: −1.805, −0.695]). This trend was not observed in females, primarily due to marked improvements in FAQLQ‐PF scores for females receiving placebo being similar in magnitude to improvements reported by females in both active treatment arms (PPOIT vs. Placebo: −0.148 [95% CI: −0.914, 0.617], OIT vs. Placebo: 0.252 [95% CI: −0.547, 1.052]). During the active treatment period analysis (T0 to T1), opposite directions of effect were observed in total score estimates between males and females when comparing PPOIT to placebo (Males: −0.514 [95% CI: −1.011, −0.017]; Females: 0.343 [95% CI: −0.322, 1.008]) and OIT to placebo (Males: −0.403 [95% CI: −0.887, 0.080]; Females: 0.517 [95% CI: −0.178, 1.212]), but not when comparing OIT to PPOIT. In other words, during treatment, males reported better HRQL improvement with active treatments compared to placebo, whereas females reported better HRQL improvement with placebo compared to either of the active treatments. In contrast, during the post‐treatment follow‐up period (T1 to T3), similar total score improvements were observed in participants of both sexes who received an active treatment compared to placebo.

**FIGURE 3:**
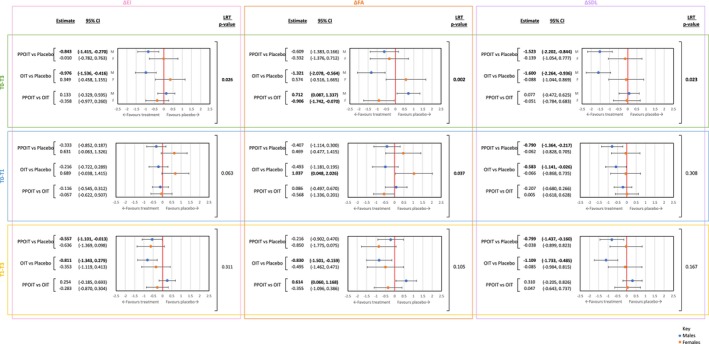
Change in FAQLQ‐PF subscores from baseline to end of follow‐up (T0 to T3), treatment (T0 to T1) and post‐treatment follow‐up (T1 to T3) analyses between treatment groups for males and females. Clinically and statistically significant mean FAQLQ‐PF change score estimates and significant LRT *p*‐values are bolded. The FAQLQ‐PF is inversely scaled: Higher scores reflect poorer HRQL. EI, emotional impact subscore; FA, food anxiety subscore; FAQLQ‐PF, Food Allergy Quality of Life Questionnaire–Parent Form; HRQL, health‐related quality of life; LRT, likelihood ratio test; OIT, oral immunotherapy; PPOIT, probiotic and peanut oral immunotherapy; SDL, social and dietary limitations subscore.

Sex‐modification of FAQLQ‐PF subdomain scores was similarly present in baseline to 12‐month post‐treatment follow‐up (T0 to T3) and baseline to end‐of‐treatment phase (T0 to T1) analyses, but not in the isolated end‐of‐treatment to 12‐month post‐treatment period (T1 to T3) (Figure 3). For the overall period (covering active treatment and post‐treatment phases), clinically meaningful improvements in all subdomain FAQLQ‐PF scores were observed in males allocated an active treatment compared to placebo. Notably, the EI change scores (PPOIT vs. Placebo: −0.843 [95% CI: −1.415, −0.270]; OIT vs. Placebo: −0.976 [95% CI, −1.536, −0.416]) and SDL change scores (PPOIT vs. Placebo: −1.523 [95% CI: −2.202, −0.844]; OIT vs. Placebo: −1.600 [95% CI: −2.264, −0.936]) of males also reached statistical significance. In contrast, as described for FAQLQ‐PF total scores, no statistically significant differences were observed in EI, FA, and SDL subscores among females allocated either PPOIT or OIT compared to placebo between T0 to T3 due to subscore improvements occurring in both active and placebo arms. During the active treatment period (T0 to T1), sex differences in treatment effect were again observed between males and females for point estimates in changes from baseline for EI and FA subscores between those receiving PPOIT or OIT compared to placebo. However, evidence for effect‐modification by sex was limited in the SDL subdomain during treatment (*p* = .308). For the isolated post‐treatment period (T1 to T3), improvements in all FAQLQ‐PF subdomains were observed in participants of both sexes allocated to PPOIT or OIT compared to placebo, though these were weaker in females than males.

**FIGURE 4:**
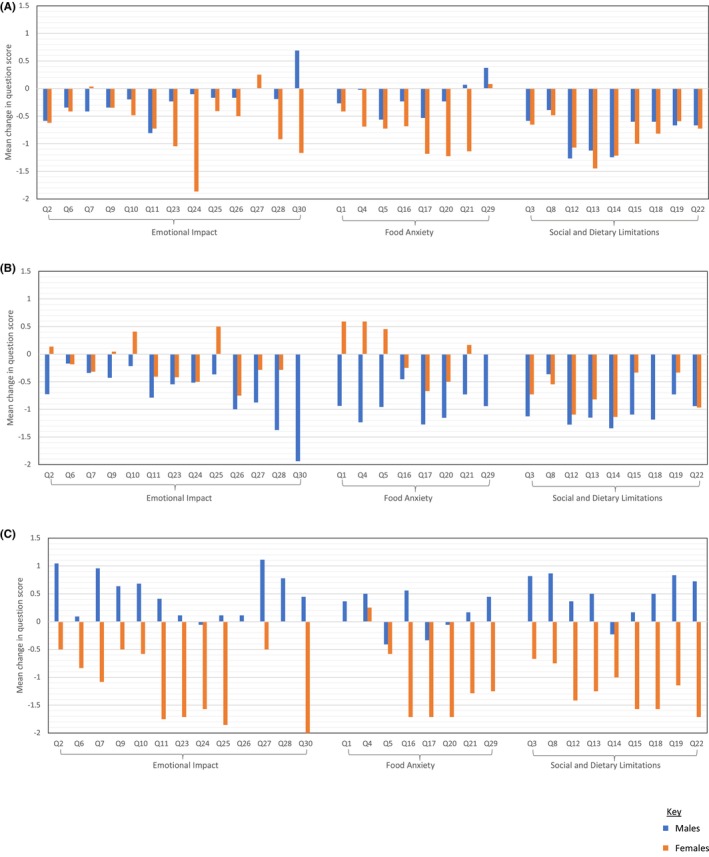
Unadjusted mean change in FAQLQ‐PF item scores between baseline to end of follow‐up (T0 to T3) in males and females per treatment group. Scores are shown for participants allocated to (A) PPOIT, (B) OIT, and (C) placebo. The FAQLQ‐PF is inversely scaled: Higher scores reflect poorer HRQL. FAQLQ‐PF items have been grouped by the HRQL subscore they contribute to. FAQLQ‐PF, Food Allergy Quality of Life Questionnaire–Parent Form; HRQL, health‐related quality of life; OIT, oral immunotherapy; PPOIT, probiotic and peanut oral immunotherapy.

When examining individual FAQLQ‐PF item score changes between baseline and 12‐months post‐treatment, most discrepancies between males and females receiving either PPOIT (Figure 4A) or OIT (Figure 4B) occurred in EI‐ and FA‐related items. The greatest difference between males and females in both PPOIT and OIT arms was seen in item 30, which asks whether a child “feels that food allergy limits his/her life in general”. In the PPOIT group, females reported HRQL improvement for item 30, while a worsening of FAQLQ‐PF score for this item was observed in males. However, in the OIT group, males reported extreme improvement in FAQLQ‐PF score for item 30 and females reported no change. Large differences in items 1 (“my child feels anxious about food”) and 4 (“my child feels afraid to try unfamiliar foods”) were also observed between males and females receiving OIT, but not those receiving PPOIT. Striking differences were seen in item change scores between males and females receiving placebo (Figure 4C). While males reported a worsening or no change across FAQLQ‐PF items, item score improvements in all but four of the FAQLQ‐PF questions were reported by females. Three items remained unchanged in females receiving placebo—items 1 (“my child feels anxious about food”), 26 (“my child wishes his/her food allergy would go away”) and 28 (“my child feels that many people do not understand the serious nature of food allergy”)—and a similar worsening in FAQLQ‐PF score for item 4 (“my child feels afraid to try unfamiliar foods”) was seen between males and females.

No evidence of bias was uncovered in sensitivity analyses. Unadjusted model coefficients were very similar to those of adjusted analyses, indicating only slightly weaker associations between treatment groups due to potential baseline imbalance (Table S1). Similar proportions of participants were lost to follow‐up from each treatment arm (Table S2).

## 4 DISCUSSION

An understanding of the influence of sex assigned at birth on OIT outcomes is needed to facilitate personalized food allergy management approaches. We investigated sex modification of treatment effects on total and subdomain FAQLQ‐PF score changes during active OIT treatment and in the 12‐month post‐treatment follow‐up period in the PPOIT‐003 study. Our findings identified sex as a potential modifier of the relationship between OIT and HRQL, notably more during active treatment than in the post‐treatment period. Sex differences were most predominant in subdomains of HRQL related to food anxiety and the emotional impact of food allergy. Sex differences could be attributed to a strong placebo response in females, where FAQLQ‐PF score improvements occurred in both OIT and placebo arms, which was not seen in males, where HRQL benefit only occurred with OIT and not placebo. We must acknowledge the explorative nature of this analysis and intend for these findings to encourage further specific research into the role of patient characteristics in food allergy treatment that is essential for improving personalized care.

The greater impact of sex on treatment effect during active therapy rather than post‐treatment follow‐up might relate to contextual factors, such as patient‐provider interactions, which have been found to contribute more to outcomes observed in randomized control trials than actual treatment.^22^ Participants have more interaction with clinical care teams during active treatment than during follow‐up, implicating psychosocial mechanisms in the observed sex‐treatment interaction.^1^, ^23^ Our finding that females allocated to placebo experienced similar HRQL improvements as those allocated to active therapy highlights that food allergy management options focused on care, counseling, or education approaches may be highly beneficial for improving the HRQL of young females and could be considered as adjunct interventions alongside immunomodulatory therapies. However, it should be noted that males did not experience HRQL improvements when allocated placebo, only when allocated OIT treatments, so the incorporation of support‐based approaches into OIT treatment plans may not benefit male children as much as it would female children.

While effect modification by sex occurred primarily during active therapy, exploration of other patient demographic characteristics, during‐treatment factors (such as adverse events and achievement of maintenance), and post‐treatment factors (such as peanut consumption and post‐treatment remission and desensitization outcomes) on HRQL is still needed to better inform personalized OIT approaches and should be prioritized in future research. A clinical outcome of remission following OIT has previously been found to be associated with greater HRQL benefit for patients than desensitization or allergic outcomes in the PPOIT‐003 trial.^24^ Our observation that similar proportions of male and female participants achieved a remission outcome (determined by end‐of‐treatment DBPCFCs) suggests the sex differences in HRQL that we observed may not be driven by differing perceptions of the clinical outcome achieved from treatment. In other words, while treatment outcome is likely to impact HRQL in general, it does not appear likely that it contributed to the sex effect modification we detected in our analysis. More extensive exploration of how post‐treatment clinical outcomes impact the relationship between OIT and HRQL would provide greater clarity here.

Sex‐disparities in EI and FA subdomain and item scores, where females again showed a placebo response not seen in male patients, reinforces our understanding that psychological factors are central drivers of HRQL.^23^ No previous speculation about whether females with food allergy may generally experience similar improvements in anxiety and emotional impact from placebo and active immunotherapy interventions has been made, though sex‐differences in related HRQL subdomains has been observed in allergic conditions at baseline.^25^, ^26^, ^27^, ^28^ Consideration of individual FAQLQ‐PF item scores suggested item 30 (“my child feels that food allergy limits his/her life in general”) was particularly influential in driving these aggregate subdomain scores observed in males and females, particularly given the stark improvement in this item for females allocated placebo and males allocated OIT. The disproportionate relief of emotional and anxiety‐related scores in females irrespective of their treatment allocation, but only in males on active treatment, might suggest that males and females experience different types of emotional stress related to their peanut allergy and consequently experience different impacts on these emotional factors during intervention.

While our results point to emotional and anxiety‐related factors as being central drivers of sex differences within the PPOIT‐003 cohort, a physiologic basis to sex differences in the placebo response has been identified in other conditions and should not be dismissed as a potential contributor to the sex differences identified in our study.^29^ Sex hormones have been suggested as key factors influencing differences in food allergy status between males and females, supported by the expression of sex hormone receptors on immune cells involved in allergic responses.^30^, ^31^, ^32^ Evidence for this assumption has been obtained in adult populations, with the onset of the menstrual cycle, pregnancy, and contraceptive use shown to alter the severity of allergy.^33^, ^34^, ^35^ However, further investigation of physiological compared to psychological drivers of food allergy outcomes in children is needed. It has also been argued that multiple interaction effects, including interactions between sex and age, may influence chronic disease‐related HRQL outcomes.^23^, ^36^ This supports our finding of an interaction between sex and treatment, but also highlights that other interaction effects need to be considered to completely understand HRQL in peanut allergy. Future investigation of multiple interaction effects is warranted to better understand HRQL discrepancies observed between males and females in this study and to facilitate optimal interventions tailored to address the different lived experiences of food allergy in males and females.

Use of a parent‐proxy instrument to measure child HRQL should also be addressed, as discrepancies between parental reporting and child self‐reporting of HRQL have been observed.^37^ Differences between child‐ and parent‐reported HRQL have been attributed to the additional burden of responsibility experienced by caregivers, especially mothers, in caring for a child with food allergy.^3^, ^38^ This suggests our results may have captured elements of the parental experience of food allergy and were therefore at risk of proxy‐induced bias.^39^ As it is unreasonable to expect young children and infants to self‐report, as was the case in this study, we hope to have mitigated the effects of proxy‐induced bias through use of the FAQLQ‐PF. This instrument is highly validated and is considered more appropriate for measurement of child allergy‐related HRQL than some child self‐report instruments, such as the Child Health Questionnaire (CHQ‐CF87).^40^ It is plausible that parent perception of child HRQL benefit may be inflated by participation in a trial, with potential overestimation of HRQL benefit by parents compared to children's self‐reports having been previously detected in a randomized controlled trial setting.^41^ This additionally may help explain some of the HRQL benefit observed in the female placebo group. However, as neither child gender identity nor parent gender identity were previously found to be influential on parent‐reported child HRQL,^5^ this alone does not help explain the sex effect modification detected in our analyses; the previously explored psychosocial and physiological drivers of HRQL in male and female children should still be considered in future research. Nonetheless, assessment of caregiver demographics and HRQL alongside child HRQL may be beneficial in future studies of young children that rely on proxy reporting to fully understand the effects of emerging treatments on food allergy‐related HRQL. Care should be taken when extrapolating results to new populations, including older children and teenagers where self‐reporting of HRQL is more plausible.

Strengths of our analysis include mitigation of the influences of baseline HRQL through adjustment in regression analysis and the observation that similar proportions of participants in each treatment arm were lost to follow‐up (see Tables S1 and S2), indicating that our results were not at serious risk of reduced validity due to baseline imbalance or missing data.^42^, ^43^ However, limited variation in participant characteristics at baseline raises the possibility of selection bias. Healthcare utilization is known to differ between SES strata, with higher rates of emergency care use but poorer representation in clinical trials being observed in those of lower SES.^44^, ^45^, ^46^ Similar to previous publications, representation of persons outside the male–female dichotomy is also lacking, which can lead to conflation of sex and gender effects.^5^ Sole consideration of sex assigned at birth, the limited sample size, as well as the majority of PPOIT‐003 trial participants being male and within high SES strata may mean that we have not captured the full spectrum of effects of treatment on HRQL. We also recognize our analysis was exploratory in nature and therefore lacks sufficient power to confirm the presence of a causal effect. Nonetheless, since no trials of OIT have yet included HRQL as a primary outcome, we must rely on post hoc analyses to extend the HRQL literature. These factors may limit the generalisability of our findings to the wider food allergy population despite the use of a multicenter trial design. Further studies involving a greater number of participants representing more diverse patient populations and specific aims to investigate HRQL as a primary outcome are needed to confirm the inferences made in this analysis.

## 5 CONCLUSION

Our findings of sex disparities in FAQLQ‐PF change scores during the treatment phase of the PPOIT‐003 trial suggest that sex assigned at birth may modify treatment effects on HRQL. An intriguing finding was that females benefitted significantly from placebo intervention primarily during the active treatment period while males did not; both males and females experienced comparable HRQL benefit from OIT. This underscores the psychological mechanisms of HRQL change and points to the application of counseling, education, and psychosocial interventions for mitigating food allergy impact, particularly in females. Further exploration of personalized approaches to care that account for sex differences in the lived experience of food allergy is warranted.

## AUTHOR CONTRIBUTIONS

**Sophie A. Rosser:** Conceptualization; methodology; formal analysis; writing – original draft; writing – review and editing; data curation; visualization. **Melanie Lloyd:** Conceptualization; methodology; supervision; writing – review and editing; data curation; formal analysis. **Paxton Loke:** Investigation; writing – review and editing. **Sarah Ashley:** Formal analysis; writing – review and editing; data curation. **Michael D. O'Sullivan:** Investigation; writing – review and editing. **Patrick Quinn:** Investigation; writing – review and editing. **Michael Gold:** Investigation; writing – review and editing. **Mimi L. K. Tang:** Conceptualization; methodology; investigation; supervision; writing – review and editing.

## FUNDING INFORMATION

The PPOIT‐003 randomized trial is an investigator‐initiated study funded by a National Health and Medical Research Council Australia Project Grant (NHMRC 115423). The Murdoch Children's Research Institute is the sponsor of the study. Additional funding support was provided by Prota Therapeutics (Melbourne, Victoria, Australia). The probiotic and placebo treatments were provided by Metagenics Australia as in‐kind support. The Murdoch Children's Research Institute is supported by the Victorian Government's Operational Infrastructure Support Program. SA Rosser is supported by the Australian Commonwealth Government through an Australian Government Research Training Program (RTP) scholarship and a PhD scholarship from the Australian Government funded National Allergy Centre of Excellence (NACE), hosted by the Murdoch Children's Research Institute (MCRI), and their work was supported by the Victorian Government's Operational Infrastructure Program.

## CONFLICT OF INTEREST STATEMENT

P Quinn reports having received grants from DBV Technologies. P Loke has received consultant fees from SPRIM Consulting and an institutional grant from Siolta Therapeutics. MLKT declares consultant fees from Pfizer and CSL Seqirus; inventorship on patents covering Peanut Oral Immunotherapy; share interest and options in Prota Therapeutics; member of the Medical Advisory Board of Anaphylaxis & Anaphylaxis Australia; member of the Board of Directors of Asia Pacific Association of Allergy Asthma and Clinical Immunology, Prota Therapeutics, and AllergyPAL; member of expert committees of the American Academy of Allergy Asthma and Immunology, Asia Pacific Association of Allergy Asthma and Clinical Immunology, and Australasian Society of Clinical Immunology and Allergy. The rest of the authors declare that they have no relevant conflicts of interest.

## PEER REVIEW

The peer review history for this article is available at https://www.webofscience.com/api/gateway/wos/peer‐review/10.1111/pai.70177.

## Supporting information


**Table S1.**pai70177‐sup‐0001‐TablesS1‐S2.pdf
