# Supplementary material for: Sex assigned at birth may modify health‐related quality of life in children treated with peanut oral immunotherapy
Source: Pediatr Allergy Immunol. 2025 Aug 22;36(8):e70177. doi: 10.1111/pai.70177 (PMC12371387; doi:10.1111/pai.70177)
Supplement: Supplementary file 1 — Table S1.pai70177‐sup‐0001‐TablesS1‐S2.pdf [file PAI-36-e70177-s001.pdf]

Supplementary material

Supplementary Table 1. Investigation of adjustment for baseline FAQLQ-PF score: comparison of adjusted and unadjusted model coefficients for the change in HRQL between T0 and T3 between treatment groups in males and females, mean (95%CI).

	PPOIT vs Placebo		OIT vs Placebo		PPOIT vs OIT	
	Males	Females	Males	Females	Males	Females
**ΔTotal**						
**Unadjusted**	-0.881 (-1.544, -0.218)*	0.086 (-0.806, 0.979)	-1.129 (-1.777, -0.481)*	0.685 (-0.242, 1.611)	0.248 (-0.288, 0.784)	-0.598 (-1.313, 0.116)
**Adjusted**	-1.003 (-1.571, -0.436)*	-0.148 (-0.914, 0.617)	-1.250 (-1.805, -0.695)*	0.252 (-0.547, 1.052)	0.247 (-0.211, 0.705)	-0.400 (-1.013, 0.213)
**ΔEI**						
**Unadjusted**	-0.729 (-1.385, -0.074)*	0.209 (-0.673, 1.092)	-0.863 (-1.504, -0.222)*	0.752 (-0.164, 1.668)	0.134 (-0.396, 0.664)	-0.543 (-1.250, 0.164)
**Adjusted**	-0.843 (-1.415, -0.270)*	-0.010 (-0.782, 0.763)	-0.976 (-1.536, -0.416)*	0.349 (-0.458, 1.155)	0.133 (-0.329, 0.595)	-0.358 (-0.977, 0.260)
**ΔFA**						
**Unadjusted**	-0.476 (-1.334, 0.382)	-0.077 (-1.232, 1.079)	-1.189 (-2.028, -0.350)*	1.045 (-0.154, 2.244)	0.713 (0.019, 1.407)*	-1.121 (-2.046, -0.196)*
**Adjusted**	-0.609 (-1.383, 0.166)	-0.332 (-1.376, 0.712)	-1.321 (-2.078, -0.564)*	0.574 (-0.516, 1.665)	0.712 (0.087, 1.337)*	-0.906 (-1.742, -0.070)*
**ΔSDL**						
**Unadjusted**	-1.398 (-2.162, -0.633)*	0.103 (-0.926, 1.133)	-1.475 (-2.223, -0.728)*	0.358 (-0.711, 1.426)	0.077 (-0.541, 0.696)	-0.254 (-1.079, 0.570)
**Adjusted**	-1.523 (-2.202, -0.844)*	-0.139 (-1.054, 0.777)	-1.600 (-2.264, -0.936)*	-0.088 (-1.044, 0.869)	0.077 (-0.472, 0.625)	-0.051 (-0.784, 0.683)

***EI**, emotional impact subscore; **FA**, food anxiety subscore; **OIT**, oral immunotherapy; **PPOIT**, probiotic and peanut oral immunotherapy; **SDL**, social and dietary limitations subscore.*
*p<0.05 for comparison between treatment groups.

Supplementary Table 2. Participants who did and did not complete the FAQLQ-PF at trial time points by treatment allocation.

	PPOIT (N = 79)	OIT (N = 83)	Placebo (N = 39)
**T0 – N (%)**			
Completed FAQLQ-PF	78 (98.73%)	83 (100.00%)	39 (100.00%)
Did not complete FAQLQ-PF	1 (1.27%)	0 (0.00%)	0 (0.00%)
**T1 – N (%)**			
Completed FAQLQ-PF	71 (89.87%)	71 (85.54%)	36 (92.31%)
Did not complete FAQLQ-PF	8 (10.13%)	12 (14.46%)	3 (7.69%)
**T3 – N (%)**			
Completed FAQLQ-PF	71 (89.87%)	69 (83.13%)	34 (87.18%)
Did not complete FAQLQ-PF	8 (10.13%)	14 (16.87%)	5 (12.82%)

FAQLQ–PF*; Food Allergy Quality of Life Questionnaire–Parent Form;* ***OIT****, oral immunotherapy;* ***PPOIT****, probiotic and peanut oral immunotherapy.*
